# Type I and type III collagen immunoexpression in rabbit skin biopsy samples treated with rosuvastatin gel and autologous platelet-rich plasma

**DOI:** 10.1590/acb402725

**Published:** 2025-03-31

**Authors:** Cristoffer da Silva Santana, Maria Rosa Santos Breda, Yuri Ferreira Vicentini, Sérgio Alexandre Alcantara dos Santos, Luis Antonio Justulin, Anthony Cesar de Souza Castilho, Gisele Alborghetti Nai, Cecília Laposy Santarém

**Affiliations:** 1 Universidade do Oeste Paulista – Postgraduate Program in Animal Science – Presidente Prudente (SP) – Brazil.; 2 Universidade do Oeste Paulista – School of Veterinary Medicine – Presidente Prudente (SP) – Brazil.; 3 Universidade Estadual Paulista – Institute of Biosciences – Botucatu (SP) – Brazil.; 4 Universidade Estadual Paulista – Institute of Biosciences – Department of Morphology – Botucatu (SP) – Brazil.; 5 Universidade do Oeste Paulista – Graduate Program in Animal Science – Presidente Prudente (SP) – Brazil.

**Keywords:** Materials Testing, Wound Healing, Skin, Hydroxymethylglutaryl-CoA Reductase Inhibitors

## Abstract

**Purpose:**

To evaluate whether the joint use of autologous platelet-rich plasma (aPRP) and rosuvastatin (RSV) in biopsies of dermal wounds induced in rabbits results in an additive effect on the immunoexpression of collagens type I and III, optimizing the healing process and increasing collagen production during the proliferative phase of healing to improve the quality of tissue repair.

**Methods:**

Thirty-two biopsy samples from eight clinically healthy adult male New Zealand rabbits were used. They were treated with aPRP, RSV, or aPRP + RSV and analyzed zero, three, seven, ten, and 14 days post wound induction.

**Results:**

Type I collagen immunoexpression was significantly higher in wounds treated with aPRP when compared to the control group. This study demonstrated that type III collagen is predominant during the proliferation phase of the healing process, highlighting its critical role in tissue repair and regeneration.

**Conclusion:**

The association of aPRP and RSV in wound treatment may have an additive effect in the immunoexpression of type III collagen and can thus be used as an alternative in tissue repair and collagen formation, optimizing the healing process.

## Introduction

Skin lesions directly interfere with life quality as they cause physical, psychological, social, professional, and economic impacts, constituting thus a serious public health issue^1^. Cortez et al.^2^ have conducted a comprehensive analysis demonstrating that advanced dressings for tissue injuries present a significantly more cost-effective alternative to conventional treatments. Their findings indicate that these advanced dressings incur costs nearly seven times lower than traditional methods, resulting in savings that exceed R$ 85,000 (approximately US$ 22,370) for the municipality.

Many alternatives have been proposed to regenerate injured tissues, but none of them are economically viable. Meanwhile, affected patients remain prone to several complicating factors, such as functional illnesses, social concerns, and economic and financial burdens^3^. A comprehensive understanding of tissue regeneration and the healing process, along with an understanding of cell–cell/biomaterial interactions^4^, is thus needed.

Activated platelets are a powerful source of growth factors that are vital for effective wound healing. One of the most innovative ways to harness their healing potential is through platelet-rich plasma (PRP). This remarkable treatment involves drawing a small amount of the patient’s venous blood and activating the platelets using substances like collagen, calcium chloride, or autologous thrombin. PRP can be delivered directly to the wound site through injections or applied topically, wherein it is often secured with a moisture-retentive dressing to create an optimal healing environment^5^.

The versatility of PRP has been demonstrated in both chronic and acute wounds, in which it has been applied at various times, depths, and frequencies. Different dosages and methods of delivery have contributed to a wide spectrum of successful healing outcomes. By choosing PRP therapy, patients can significantly enhance their healing process and get back to their everyday lives faster^6^.

Statins are a class of drugs that are well-known for their lipid-lowering effect; accordingly, they are generally given for the treatment of cardiovascular diseases^7^. Research has shown their ability to manage conditions other than heart problems, such as allergic inflammation^8^ and wound healing^9^^,^ 10. Rosuvastatin (RSV) is one of the statins that lowers low-density lipoprotein and raises high-density lipoprotein by inhibiting 3-hydroxy3-methyl glutaryl co-enzyme A (HMG-COA) reductase^11^.

RSV was experimentally proven to be effective in wound healing as it reverses the effect of the inhibitors of wound healing, such as farnesyl pyrophosphate, and stimulates microvascular and endothelial functions, which enhance wound healing processes. In addition, it interferes with the synthesis of selective proteins in bacteria by blocking many cellular processes and biosynthetic pathways. This increases its capability to stop the formation of key methicillin-resistant *Staphylococcus aureus* toxins, which delay the growth of septic skin lesions^10^.

In a study by Maged et al.^12^, RSV was loaded in chitosan scaffolds to be applied topically for wound healing which showed enhanced skin healing and regeneration. Besides these protective and pro-healing factors, RSV aided in the expression of fibers that contribute to the integrity of tissues, such as collagen.

The topical route has many advantages over the oral route in wound healing for many reasons, such as avoiding drug degradation in the liver, lowering the systemic side effects, easy application, overage of large surface areas of the body^13^, accelerating healing and reduced resistance of bacteria^14^.

Considering the importance of autologous PRP (aPRP) as a promising biomaterial and the few studies involving a statin in the healing process, the present study aimed to evaluate whether the joint use of aPRP and RSV in biopsies of dermal wounds induced in rabbits results in an additive effect on the immunoexpression of collagens type I and III, optimizing the healing process and increasing collagen production during the proliferative phase of healing to improve the quality of tissue repair.

## Methods

The study was approved by the Animal Use Ethics Committee of the Universidade do Oeste Paulista, in Presidente Prudente, São Paulo, Brazil (protocol no. 5,170).

### Experimental design and animals

The minimum sample size was determined using the function pwrss.f.rmanova from the pwrss package in R^15^. For this calculation, a large effect size for the treatment was considered, specifically Cohen’s d = 0.40, which pertains to the percentage of type II collagen. Additionally, a correlation of 0.50 between pre-test and post-test scores was assumed, corresponding to η^2^ = 0.242. The parameters set for the study included an experimental group without a control, four repetitions, a test power of 80%, and a significance level of 5%. Based on this, it was concluded that a minimum of six animals was required to conduct the study.

Eight 2-year-old, clinically healthy, adult New Zealand male rabbits weighing 3 ± 1 kg received treatment with aPRP, RSV, or an association of both. The animals were kept in individual cages under room temperature (22°C ± 2°C) and controlled photoperiod (12-h light/dark). The rabbits went through an adaptation period of seven days prior to commencing the study. Throughout the experiment, they were kept under standardized conditions of diet and water *ad libitum*.

### Anesthetic procedure

First, rabbits were handheld for the right and left dorsal trichotomy and, then, they were anaesthetized (intramuscular) with a combination of xylazine (Xylazine 2%, 5 mg/kg) and tiletamine hydrochloride and zolazepam (Zoletil 50, 15 mg/kg)^16^.

### Autologous platelet-rich plasma

**Figure 1:**
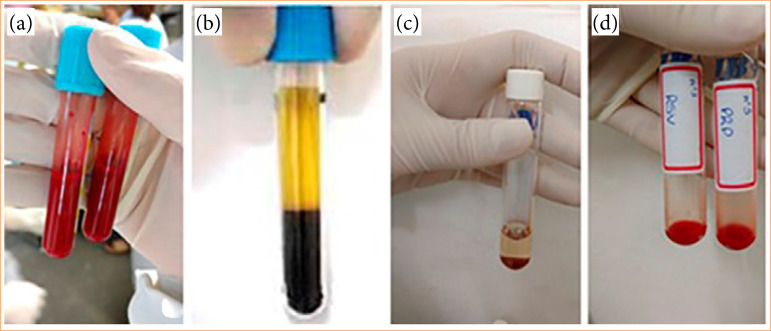
Autologous platelet-rich plasma (aPRP) procedure. **(a)** Tubes containing sodium citrate anticoagulant filled with blood collected from the rabbit’s auricular vein. **(b)** Appearance of the blood after the first centrifugation at 200 G. **(c)** Tube after the second centrifugation at 400 G. **(d)** Tubes containing aPRP and aPRP mixed with rosuvastatin, both transformed into gel form and ready for application to the wounds.

Source: Elaborated by the authors.

After an anesthetic procedure, 4 mL of venous blood was collected from each rabbit’s ear using a 25-G scalp. The material was transferred to a flask containing an anticoagulant (sodium citrate). An aliquot of this solution was used for automatic platelet counting (Sysmex pocH-100i Automated Hematology Analyzer) and then centrifuged (Excelsa Baby 206R centrifuge) at 200 g for 10 min to form two layers: the entire fraction corresponding to the plasma, and 200 μL of the red-colored layer. The content from the latter was transferred to another tube for additional centrifugation at 400 g for 10 min^9^. This technique enables the recovery of the platelet and leukocyte fraction (Fig. 1).

### Rosuvastatin gel

To prepare the gel, 105 mg of methylcellulose was combined with 5 mL of distilled water heated to a temperature between 50 and 60°C. The mixture was stirred continuously until the gel formed, which was designated as solution A. Meanwhile, 84 mg of RSV was dissolved in 2 mL of distilled water and stirred to create solution B. Next, solution A and solution B were mixed while stirring to ensure a homogeneous gel and avoid crystallization. The final solution contained 1.2 mg of RSV in every 0.1 mL of the gel^17^.

### Wound induction and treatment

**Figure 2:**
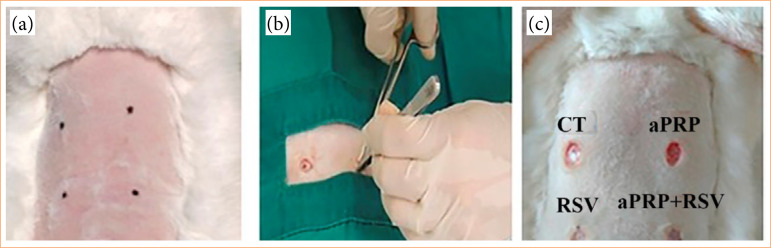
Induction and treatment of injuries. **(a)** A rabbit’s back is marked with a Pilot pen to indicate the area for a future wound induction. **(b)** A fragment of skin is being removed using tweezers and surgical scissors. **(c)** The dorsal of the rabbit with wounds that were created using an 8-mm punch.

With the rabbits under anesthesia, the skin on their dorsum was disinfected and marked, and surgical wounds were inflicted in four equidistant places using a pen and an 8-mm punch. The skin fragments were removed using anatomical forceps, preserving the musculature. Each wound received different treatments: saline solution (control treatment), aPRP, RSV, or aPRP + RSV. The RSV gel was manually applied to wounds treated with RSV alone or associated with aPRP, using enough to cover the lesion^9^ (Fig. 2).

CT: 0.9% saline solution; aPRP: autologous platelet-rich plasma; RSV: rosuvastatin. Source: Elaborated by the authors.

All wounds were subsequently covered with sterile rayon and adhesive bandage (Band Aid). After the surgical procedure, the animals received tramadol hydrochloride (0.5 mg/kg, intramuscular) twice a day for three consecutive days to minimize initial discomfort.

Treatment and dressing changes were performed on days 0, 3, 7, 10, and 14 post wound induction following the protocol of Vendramin et al.^18^ until 16 days of experiment were completed. On the 17^th^ day, biopsy samples of the wounds were evaluated.

The animals were anaesthetized with a combination of tiletamine hydrochloride and zolazepam (30 mg/kg intramuscular) and xylazine at 2% (0.2 mg/kg intramuscular) and euthanized in a CO_2_ chamber. Proof of death was based on heart rate. After euthanasia, the animals were identified, frozen, and stored for use in practical classes.

### Immunohistochemical analysis

Histological verification of type I and type III collagens in the healing process on day 17 was performed using the indirect immunoperoxidase technique. Antibodies for evaluating collagen immunoexpression in rabbit wounds treated with aPRP, RSV, and aPRP + RSV included: anti-type I collagen primary antibody (1:50, mouse monoclonal, GeneTex, GTX26308), anti-type III collagen primary antibody (1:100, mouse monoclonal, Novus Biologicals, NBP1-05119), and goat anti-mouse IgG H&L (HRP) antibody (1:200, goat, Abcam, ab6789). This procedure was performed at the Extracellular Matrix Laboratory of the Morphology Department of the Botucatu Biosciences Institute of the Universidade Estadual Paulista “Júlio de Mesquita Filho” in Botucatu, São Paulo, Brazil.

Thirty-two skin samples were used, eight from each group (control, aPRP, RSV, and aPRP + RSV). These samples were fixed in 10% formalin solution, buffered at pH 7.0 for 24–48 h, and then washed in running water for 1 h. They were then transferred to a 70% alcohol solution. After performing standardized procedures for embedding paraffin tissues, 4-μm slices were obtained and mounted on silanized slides. For each sample, three slides were analyzed.

For antigenic recovery, the slides were immersed for 30 min in citrate buffer (pH 6.0) in a pressure cooker (approximately 100°C) in a water bath. Slides were then washed with phosphate buffered saline (PBS) and subjected to the process of blocking endogenous peroxidase using a mixture of PBS and hydrogen peroxide for 10 min in the dark. Then, another block procedure was undertaken using 5% skimmed milk in PBS.

The sections were incubated overnight at 4°C with anti-type I or anti-type III collagen primary antibodies diluted in 1% BSA. Subsequently, the slides were washed with PBS. The skin sections were incubated with the secondary antibody, also diluted in 1% BSA, for 1,5 h at room temperature. The immunohistochemical reaction was then revealed with diaminobenzidine (DAB).

As enough collagen is present in the skin, no positive control was used. Moreover, one of the cuts was not incubated with primary antibody (only the secondary antibody) to be used as negative control. Slides were subsequently counterstained with hematoxylin. All dilutions used had been previously tested^19^.

### Image acquisition

Images were acquired using an optical microscope (Leica DMLB, São Paulo, SP, Brazil) coupled to a camera (Leica DFC300 FX, São Paulo, SP, Brazil). The images observed under the microscope were projected on a monitor using an image analysis software (LeicaQWin Plus, São Paulo, SP, Brazil). Images from the region below the epidermis were obtained at a 400x magnification.

The immunostained area, which showed a brownish color, was evaluated using the color deconvolution technique via the “Colour Deconvolution” plugin in the Fiji software. For this, the image was separated into three colors: green (bottom of the slide), blue (hematoxylin), and brown (immunostained area), the latter being used for quantification. In the “Images” section of the software, “Threshold” was used for the selection of the marked areas and subsequent quantification. To avoid the detection of insufficient staining in heavily stained areas or excessive staining in poorly stained areas, a range of 0–180 was established for the brown tones of the histogram. After applying the mask to the immunostained area, the percentage of this staining in relation to the area was measured, and an average of three images was calculated^20^.

### Statistical analysis

The statistical evaluation of the effect of treatments was performed using the GraphPadPrism software, version 5.01. Data normality was assessed via Shapiro-Wilk’s test, and homogeneity of variances using Bartlett’s test. For comparison between treatments, Kruskal-Wallis’ and Tukey’s tests were performed. Differences were considered significant when *p* < 0.05.

## Results

**Figure 3:**
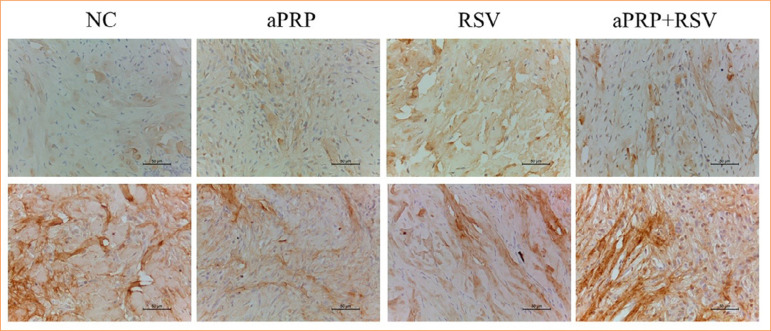
Immunohistochemical analysis of the lesions. **(a)** Immunohistochemical staining for type I collagen on day 17 in the NC and the different treatments. Magnification: 400x. Scale bar: 50 μm. **(b)** Immunohistochemical staining for type III collagen on day 17 in the NC and the different treatments.

NC: negative control; aPRP: autologous platelet-rich plasma; RSV: rosuvastatin. Source: Elaborated by the authors.

**Figure 4:**
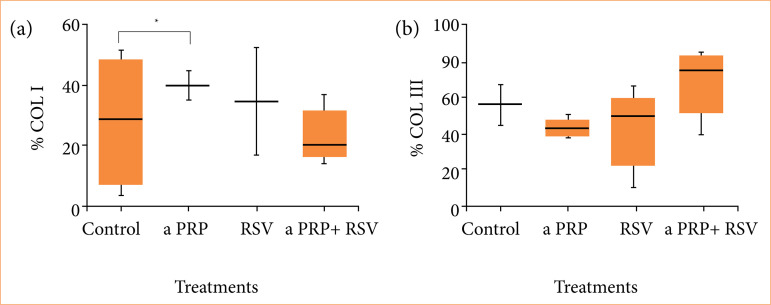
Plots representing the average percentage of immunostaining for types I and III collagen under different treatments on day 17.

NC: negative control; aPRP: autologous platelet-rich plasma; RSV: resovastatin; *p < 0.05. Source: Elaborated by the authors.

Type I collagen expression was higher (*p* < 0.05) in animals treated with aPRP alone (39.9 ± 4.6) and in those receiving aPRP in combination with RSV (45.0 ± 3.7) compared to control wounds (28.0 ± 1.7) on day 17. The expression of RSV alone (34.4 ± 1.8) did not differ statistically from the other groups. For type III collagen expression (*p* > 0.05), the observed mean values were as follows (Figs. 3 and 4):

- Control: 56.6 ± 1.1;
- aPRP: 43.2 ± 3.4;
- RSV: 42.7 ± 1.6;
- aPRP + RSV: 68.7 ± 1.4.

## Discussion

We here in demonstrated that the aPRP + RSV treatment led to a predominance of type III collagen, i.e., the highest immunoexpression of this type of collagen and the lowest immunoexpression of type I collagen. In contrast, the aPRP treatment yielded greater immunoexpression of type I collagen compared to the other treatments.

According to Witte and Barbul^21^, type III collagen predominates over type I collagen during the inflammation phase of the healing process, whereas type I collagen predominates in the following phases (proliferation and maturation). However, in the present study, the immunoexpression of type III collagen predominated over that of type I in the aPRP + RSV treatment during the putative proliferation phase, which may indicate that the wounds were in fact still in the healing phase^22^.

Pietrovski et al.^23^ reviewed the use of statins in the treatment of diabetic foot ulcers and found that experimental studies suggested the application of statins to this type of injury as they may favor the healing process. Despite this evidence on statins accelerating the healing of skin lesions, further clinical studies are needed to evaluate this therapeutic use.

Bacteria present in wounds impair healing by activating the alternative complement pathway, amplifying, and prolonging the inflammatory phase of healing. Shen et al.^24^ demonstrated that continuous inflammation resulting from infection significantly impairs tissue repair in zebrafish larvae. This research expands on previous work that established a link between inflammatory macrophages and impaired wound healing. Ko et al.^25^ evaluated the antibacterial effects of statins on bacterial pathogens that cause *in-vitro* skin infections and found that statins can reduce biofilm formation and decrease bacterial adhesion to environmental surfaces. Olivetti et al.^26^ used modified collagen hydrogels impregnated with simvastatin in skin wound dressings and observed that the antibacterial activity was prolonged, thus confirming the antimicrobial properties of statins. Moreover, Cieślik-Bielecka et al.^27^ demonstrated that leukocyte-rich aPRP exhibits bacteriostatic activities against *S. aureus, Enterococcus faecalis* and *Pseudomonas aeruginosa*. These results emphasize the importance of infection control in the healing process. In our study, the induced wounds remained clean and free of signs of infection throughout the study and treatment periods.

Maged et al.^12^ used mesenchymal stem cells associated with chitosan dressings impregnated with RSV in albino rats to improve the healing of induced wounds; they concluded that RSV-impregnated wound dressings increased the proliferation of dermal fibroblasts compared to the placebo. Moreover, 30 days after the implantation of this dressing, they observed an improved dermal regeneration and absence of hypertrophic scar formation. In our study, the evaluation took place 17 days after injury infliction, thus hindering the observation of the healing process throughout a longer period. Therefore, further studies are necessary to assess tissue repair in the following phases.

Ferreira et al.^28^ studied the effect of combining biomaterials and topical RSV on tissue repair in surgical wounds in rabbits. The biomaterials used were aPRP or aPRF alone or in association with RSV. Their results showed that RSV alone and aPRF + RSV induced a significantly higher production of fibroblasts compared to the control and aPRF treatments. The authors concluded that both RSV and aPRF exhibited good healing effects, and that the combination of both was beneficial and demonstrated a possible additive effect in the proliferation of fibroblasts, corroborating our study in the association of a biomaterial with RSV to improve general aspects of the healing process.

Few studies have specifically evaluated the use of aPRP and RSV in wound healing, especially considering the immunoexpression of different types of collagens. Tetila et al.^9^ conducted a longitudinal study on the use of aPRP and RSV in wound healing in rabbits with the assessment of microscopic and macroscopic aspects. These authors observed that aPRP + RSV and aPRP alone yielded a higher amount of collagen fibers (90.76 and 90.07%, respectively) when compared to RSV alone (85.98%) and the control group (78%). These results corroborate those of our study on the increase of collagen fibers by using aPRP alone or in association with RSV. Furthermore, wounds treated with aPRP resulted in edges with more homogeneous closure, reaffirming the importance of biomaterials in wound healing.

In clinical practice, the biological activities of biomaterials combined with statins could play a significant role in treating skin lesions, such as diabetic ulcers, as well as other hard-to-heal wounds that may show signs of infection. The authors conducted their study on healing over a period of only 17 days following injury, which means they did not assess later phases, including long-term tissue maturation and repair. Additionally, since this was an experimental study with measures in place to control infections–a common complication in real-world wound healing–, the findings cannot be used to evaluate how the treatments would perform in the presence of infected wounds.

## Conclusion

It was concluded here that the association of aPRP + RSV in wound treatment exhibited a possible additive effect in the immunoexpression of type III collagen and can therefore constitute a viable alternative for tissue repair and collagen formation, optimizing the healing process.

## Data Availability

The data will be available upon request.
